# Early Prone Positioning in Three Pediatric Cases of Post-drowning Acute Respiratory Failure: A Case Series of Short-Term Changes in Oxygenation and Respiratory System Compliance

**DOI:** 10.7759/cureus.102778

**Published:** 2026-02-01

**Authors:** Tamotsu Gotou, Takahiro Hagihara, Yamato Wada, Kyoji Hashimoto, Futoshi Nagashima

**Affiliations:** 1 Department of Pediatric Emergency and Critical Care Medicine, Tottori Prefectural Central Hospital, Tottori, JPN; 2 Department of Emergency and Critical Care Medicine, Tottori Prefectural Central Hospital, Tottori, JPN; 3 Department of Emergency Medicine, Toyooka Hospital, Toyooka, JPN

**Keywords:** acute respiratory failure, mechanical ventilation, near-drowning, pediatrics, pediatrics intensive care, prone positioning

## Abstract

Drowning is a process of respiratory impairment resulting from submersion or immersion in a liquid, and severe cases may develop acute respiratory failure due to drowning-associated lung injury. Prone positioning is a recognized adjunctive therapy for acute respiratory distress syndrome (ARDS), yet evidence in pediatric drowning remains limited. We conducted a retrospective, descriptive case series of three pediatric seawater drowning patients (age 2-6 years) without cardiac arrest who required endotracheal intubation and invasive mechanical ventilation and received early prone positioning for 2-4 hours. The primary descriptive outcomes were short-term changes (within four hours) in oxygenation (arterial oxygen partial pressure (PaO_2_)/fraction of inspired oxygen (FiO_2_) ratio, or P/F ratio) and dynamic respiratory system compliance normalized to body weight (Cdyn/kg). The pre-prone P/F ratio ranged from 158 to 250 and increased to 316-506 within four hours after prone positioning was initiated. Cdyn/kg (defined as tidal volume divided by the difference between peak inspiratory pressure and positive end-expiratory pressure (PEEP), normalized to body weight) increased from 0.45-0.80 to 0.88-1.00 mL/cmH_2_O/kg. The duration of mechanical ventilation was 20-23.5 hours, and the intensive care unit (ICU) length of stay was 2-3 days. All patients survived to discharge without sequelae. These observations are hypothesis-generating, and larger studies are needed to clarify efficacy, indications, and optimal conditions of early, short-duration prone positioning in pediatric drowning-associated acute respiratory failure.

## Introduction

Drowning is defined as a process of respiratory impairment from submersion or immersion in a liquid and encompasses both fatal and non-fatal events [1,2]. Terms such as “near-drowning” are no longer recommended internationally because they promote inconsistent definitions [1,2]. In children, drowning remains a leading cause of preventable injury-related death, and survivors frequently develop respiratory failure [3,4].

Post-drowning respiratory failure is driven by aspiration and alveolar washout, resulting in surfactant dysfunction, increased alveolar-capillary permeability, atelectasis, and increased intrapulmonary shunt, which can progress to pulmonary edema and an acute respiratory distress syndrome (ARDS)-like phenotype [4,5]. Accordingly, post-resuscitation respiratory management should be grounded in lung-protective ventilation (appropriate positive end-expiratory pressure (PEEP) and avoidance of overdistension), with stepwise consideration of adjunctive therapies for refractory hypoxemia [5,6].

Prone positioning improves oxygenation through recruitment of dependent dorsal lung regions and improved ventilation-perfusion matching and has been shown to improve outcomes in adults with severe ARDS [7]. In pediatric acute lung injury/pediatric acute respiratory distress syndrome (PARDS), randomized controlled trials suggest that prone positioning can improve oxygenation, but consistent benefits in clinical outcomes such as ventilator-free days have not been demonstrated [8,9]. Observational studies, however, have reported improved oxygenation with early and repeated prone positioning, and the role of positional therapy in children remains an area of ongoing investigation [10].

A systematic review of therapeutic interventions for drowning-associated lung injury highlighted the scarcity of clinical trials and the difficulty of determining effectiveness based on comparative evidence [11]. Reports of prone positioning in drowning are largely limited to isolated case reports, including use in spontaneously breathing patients and early prone positioning combined with lung-protective ventilation in near-fatal drowning, while systematic evaluation is lacking [12,13].

Although the optimal timing of prone positioning in drowning-associated lung injury has not been standardized, Lee et al. reported a drowning case complicated by ARDS and multi-organ failure in which prone mechanical ventilation was initiated after hemodynamic stabilization, approximately two hours after intensive care unit (ICU) admission, with rapid improvement in oxygenation within two hours of repositioning [14].

Here, we present a retrospective, descriptive case series of three pediatric patients with acute respiratory failure following non-cardiac arrest drowning who received early prone positioning - defined as initiation within two hours of ICU admission - and short-duration prone sessions (2-4 hours) during invasive mechanical ventilation.

## Case presentation

Study design and data collection

This study was a single-center, retrospective, descriptive case series based on electronic medical records. We included three non-cardiac-arrest pediatric patients who, since 2017, required endotracheal intubation and invasive mechanical ventilation for acute respiratory failure after drowning and subsequently underwent early prone positioning therapy, initiated within two hours of ICU admission. Mechanical ventilation was delivered in all cases using pressure-controlled assist/control (PCV-A/C). Sedation and analgesia during invasive mechanical ventilation consisted of continuous midazolam and fentanyl infusions in all patients. During prone positioning sessions, continuous neuromuscular blockade was administered. Respiratory and hemodynamic monitoring during prone positioning included continuous ECG, pulse oximetry (peripheral oxygen saturation (SpO_2_)), end-tidal carbon dioxide (EtCO_2_), and invasive arterial blood pressure monitoring via an arterial line.

Variables

From the electronic medical records, we extracted age (years), body weight (kg), drowning circumstances (location, estimated submersion time, and prehospital responses/interventions), Szpilman drowning severity classification [15], initial venous blood gas results, arterial blood gas results at prespecified time points, ventilator parameters (peak inspiratory pressure, PEEP, fraction of inspired oxygen (FiO_2_), and tidal volume), and duration of prone positioning (hours). We calculated the arterial oxygen partial pressure (PaO_2_)/FiO_2_ ratio, or P/F ratio, and derived indices of respiratory mechanics, including dynamic respiratory system compliance (Cdyn = tidal volume/(peak inspiratory pressure - PEEP)) and weight-normalized compliance (Cdyn/kg, defined as Cdyn divided by body weight). We also recorded the duration of mechanical ventilation (hours), length of stay in the ICU (days), and clinical outcomes.

Outcomes and assessment time points

Before data extraction, we predefined the primary descriptive outcomes as changes in the P/F ratio and Cdyn/kg from immediately before initiation of prone positioning to approximately four hours after initiation. This time window was selected to capture early physiological responses during the initial prone session while minimizing the influence of subsequent changes in clinical course and ventilator management. Secondary outcomes included duration of mechanical ventilation, ICU length of stay, and survival to discharge without neurological sequelae. During the observation window (immediately before prone positioning and within four hours after initiation), PEEP was intentionally kept unchanged in all patients to minimize potential confounding due to changes in ventilatory settings. FiO_2_ was titrated according to arterial oxygenation (PaO_2_) at the discretion of the treating physicians; therefore, oxygenation was primarily summarized using the P/F ratio, which inherently accounts for changes in FiO_2_. Apart from prone positioning, no other major therapeutic interventions were introduced during the observation period. Peak inspiratory pressure, PEEP, and tidal volume were recorded as the mean of three consecutive respiratory cycles measured at each time point. Dynamic respiratory system compliance (Cdyn/kg) was calculated exclusively from controlled breaths under neuromuscular blockade, using ventilator-measured tidal volume, peak inspiratory pressure, and PEEP (not preset settings), with no spontaneous breathing efforts at the measurement time points.

Case 1

**Figure 1:**
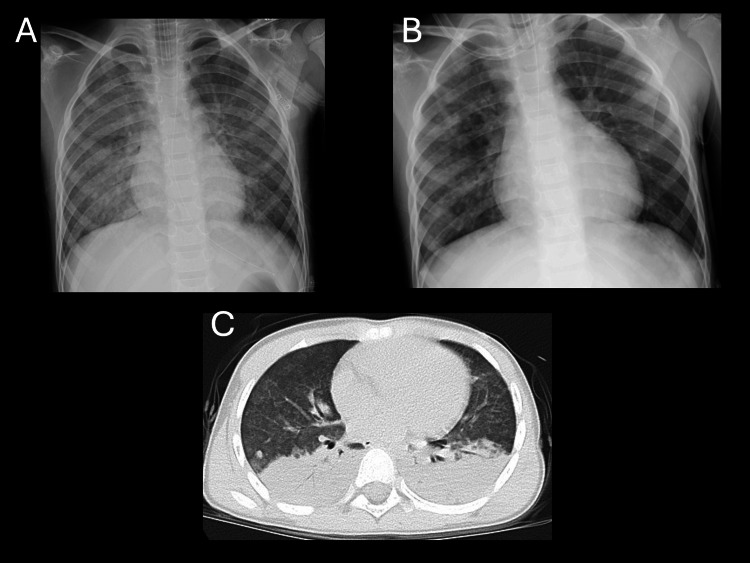
Chest radiographs and chest CT images in case 1 (A) Chest radiograph obtained before prone positioning. (B) Chest radiograph obtained after prone positioning, demonstrating improved lung radiolucency compared with panel A. (C) Chest CT image obtained before prone positioning, showing extensive dorsal atelectasis. Note: The left side of each image corresponds to the patient’s right side.

A six-year-old boy (21.3 kg) experienced seawater drowning with an estimated maximum submersion time of 10 minutes. Prehospital care via physician-staffed helicopter included endotracheal intubation using a cuffed tube (Microcuff®, Avanos Medical, Alpharetta, Georgia, USA) with an internal diameter of 5.0 mm, secured at 15 cm at the right corner of the mouth, followed by transport to our hospital, where mechanical ventilation was continued. Arterial blood gas analysis obtained immediately before prone positioning showed pH 7.15, base excess -8.8 mmol/L, and lactate 4.8 mmol/L. The Szpilman drowning grade was 3 [15]. Immediately after intubation, the P/F ratio was 250 (PEEP 10 cmH_2_O, FiO_2 _0.5), and prone positioning was initiated early for two hours. Four hours after starting prone positioning, the P/F ratio increased to 462 (PEEP 10 cmH_2_O, FiO_2_ 0.4), and Cdyn/kg increased from 0.80 to 1.00 mL/cmH_2_O/kg. Pre-prone chest radiograph and CT, as well as post-prone chest radiograph, are shown in Figure 1. The patient was extubated 23.5 hours after ICU admission. He stayed in the ICU for two days and was discharged home on hospital day four without sequelae.

Case 2

**Figure 2:**
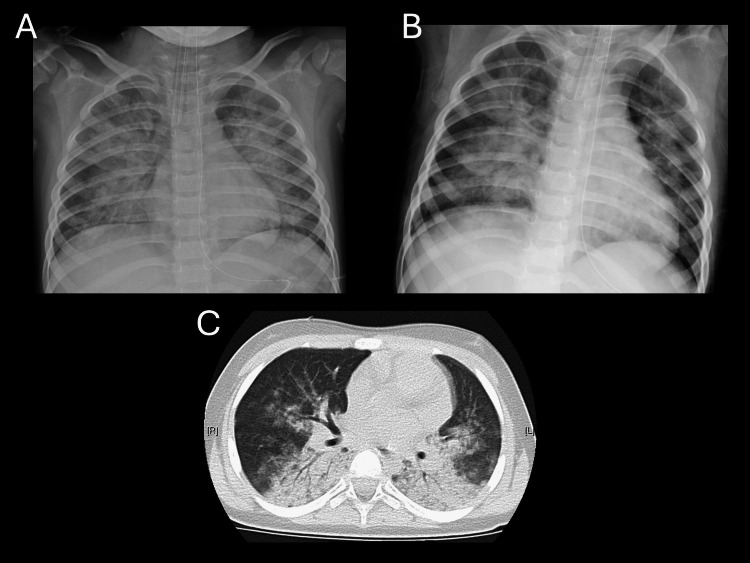
Chest radiographs and chest CT images in case 2 (A) Chest radiograph obtained before prone positioning. (B) Chest radiograph obtained after prone positioning, showing slight improvement in lung radiolucency compared with panel A. (C) Chest CT image obtained before prone positioning, demonstrating extensive dorsal atelectasis. Note: The left side of each image corresponds to the patient’s right side.

A two-year-old boy (15.4 kg) experienced seawater drowning with an estimated maximum submersion time of five minutes. Prehospital intubation was performed via physician-staffed helicopter using a cuffed tube (Microcuff®, Avanos Medical, Alpharetta, Georgia, USA) with an internal diameter of 4.0 mm, secured at 12 cm at the right corner of the mouth, and mechanical ventilation was continued after arrival. Arterial blood gas analysis obtained immediately before prone positioning showed pH 7.03, base excess -12.9 mmol/L, and lactate 2.1 mmol/L. The Szpilman drowning grade was 3 [15]. Immediately after intubation, the P/F ratio was 222 (PEEP 10 cmH_2_O, FiO_2 _0.6), and prone positioning was initiated early for two hours. Four hours after starting prone positioning, the P/F ratio increased to 506 (PEEP 10 cmH_2_O, FiO_2_ 0.4), and Cdyn/kg increased from 0.70 to 0.97 mL/cmH_2_O/kg. Pre-prone chest radiograph and CT, as well as post-prone chest radiograph, are shown in Figure 2. The patient was extubated 22.5 hours after ICU admission. ICU length of stay was three days. Because he reported transient visual disturbance, his hospitalization was extended for follow-up; once the symptoms resolved, he was discharged on hospital day 15 without sequelae.

Case 3

**Figure 3:**
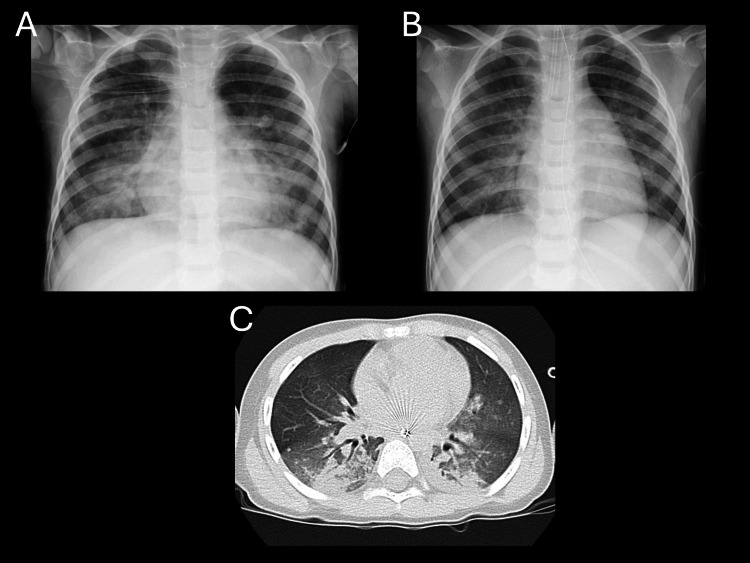
Chest radiographs and chest CT images in case 3 (A) Chest radiograph obtained before prone positioning. (B) Chest radiograph obtained after prone positioning, demonstrating improved radiolucency in the lower lung fields compared with panel A. (C) Chest CT image obtained before prone positioning, showing dorsal atelectasis. Note: The left side of each image corresponds to the patient’s right side.

A three-year-old boy (13.2 kg) experienced seawater drowning with an estimated maximum submersion time of five minutes. No prehospital advanced care was provided. After arrival, he was intubated with a cuffed tube (Microcuff®, Avanos Medical, Alpharetta, Georgia, USA) with an internal diameter of 4.5 mm, secured at 14 cm at the right corner of the mouth, and invasive mechanical ventilation was initiated. Arterial blood gas analysis obtained immediately before prone positioning showed pH 7.27, base excess -6.4 mmol/L, and lactate 0.8 mmol/L. The Szpilman drowning grade was 2 [15]. Immediately after intubation, the P/F ratio was 158 (PEEP 10 cmH_2_O, FiO_2 _0.7), and prone positioning was initiated early for four hours. Four hours after starting prone positioning, the P/F ratio increased to 316 (PEEP 10 cmH_2_O, FiO_2 _0.4), and Cdyn/kg increased from 0.45 to 0.88 mL/cmH_2_O/kg. Pre-prone chest radiograph and CT, as well as post-prone chest radiograph, are shown in Figure 3. The patient was extubated 20 hours after ICU admission. ICU length of stay was three days, and he was discharged on hospital day four without sequelae.

Summary of cases

A summary of the three cases is presented in Table 1. Across all three cases, both the P/F ratio and weight-normalized dynamic compliance (Cdyn/kg) increased from baseline to the four-hour assessment.

**Table 1:** Baseline characteristics, drowning severity, and short-term changes in oxygenation and dynamic compliance in three pediatric drowning cases managed with prone positioning Estimated submersion time indicates the maximum estimated duration. Arterial pH, base excess, and lactate were obtained from arterial blood gases drawn immediately before prone positioning and approximately four hours after initiation of prone positioning; these values correspond to the same time points as the P/F ratios. P/F ratios were calculated from arterial blood gases obtained immediately before prone positioning (P/F before prone), approximately four hours after initiation of prone positioning (P/F after prone), and immediately prior to extubation (P/F before extubation). Dynamic compliance per kilogram (mL/cmH_2_O/kg) was calculated as tidal volume divided by the difference between peak inspiratory pressure and PEEP, normalized to body weight; values correspond to the same time points as the P/F ratios. Drowning grade was assessed using the Szpilman classification [15]. ICU: intensive care unit; P/F: arterial oxygen partial pressure (PaO_2_)/fraction of inspired oxygen (FiO_2_); PEEP: positive end-expiratory pressure

Case	Age (years)	Weight (kg)	Location	Estimated submersion time (min)	Prehospital intubation	Duration of prone positioning (hr)	pH before prone	Base excess before prone (mmol/L)	Lactate before prone (mmol/L)	pH after prone	Base excess after prone (mmol/L)	Lactate after prone (mmol/L)	P/F before prone	P/F after prone	P/F before extubation	Dynamic compliance before prone (mL/cmH_2_O/kg)	Dynamic compliance after prone (mL/cmH_2_O/kg)	Duration of mechanical ventilation (hr)	ICU length of stay (days)	Drowning grade	Outcome
1	6	21.3	Sea	10	Yes	2	7.15	-8.8	4.8	7.38	-1.8	1.0	250	462	542	0.8	1.0	23.5	2	3	No sequelae
2	2	15.4	Sea	5	Yes	2	7.03	-12.9	2.1	7.28	-6.7	1.6	222	506	433	0.7	0.97	22.5	3	3	No sequelae
3	3	13.2	Sea	5	No	4	7.27	-6.4	0.8	7.29	-4.0	0.7	158	316	350	0.45	0.88	20.0	3	2	No sequelae

## Discussion

In this retrospective, descriptive case series of three pediatric patients with post-drowning acute respiratory failure without cardiac arrest, we observed increases in oxygenation (P/F ratio) and respiratory mechanics (Cdyn/kg) within four hours after initiation of early, short-duration prone positioning (2-4 hours) during invasive mechanical ventilation. Because this report is non-comparative and bedside management (including ventilator settings) may have been adjusted concurrently, these observations should be interpreted as descriptive and hypothesis-generating rather than evidence of efficacy.

In drowning-associated lung injury, aspiration and alveolar washout lead to surfactant dysfunction, increased permeability, and atelectasis, with dependent dorsal lung collapse and increased shunt as major drivers of hypoxemia [4,5]. Prone positioning alters thoracic mechanics and gravitational effects, improves ventilation of dorsal lung regions, and mitigates ventilation-perfusion mismatch, which may improve oxygenation [7]. Notably, we observed concurrent increases not only in oxygenation but also in Cdyn/kg, which may be consistent with alveolar recruitment and more homogeneous ventilation.

In a randomized trial by Kornecki et al. in pediatric acute lung injury, prone positioning for two hours improved oxygenation, whereas significant changes in compliance were not reported [9]. In a multicenter randomized trial by Curley et al., prone positioning (mean ~20 hours/day) did not improve clinical outcomes such as ventilator-free days [8]. The short-term increases in Cdyn/kg observed in our case series could reflect a larger proportion of recruitable lung early after drowning, with potentially reversible atelectasis and pulmonary edema; however, the small sample size and non-comparative design preclude inference regarding treatment effect.

A systematic review of interventions for drowning-associated lung injury reported therapies such as surfactant, inhaled nitric oxide, and extracorporeal membrane oxygenation (ECMO) but noted limited comparative evidence and low overall certainty [11]. For prone positioning, published evidence in drowning remains largely case-based, including reports in spontaneously breathing patients and early proning combined with lung-protective ventilation in near-fatal drowning [12,13]. Although a multicenter, retrospective cohort has examined respiratory management in drowning-associated acute respiratory failure, pediatric-specific criteria for selecting patients for proning, optimal timing, and duration remain unclear [16].

We used the Szpilman grading system to assess drowning severity and report outcomes [15]. Beyond providing a standardized description of clinical severity, Szpilman grading may help contextualize the clinical trajectory and support comparability across studies and case reports. In our series, all three patients were graded as Szpilman grade 2 or 3 (moderate to severe respiratory impairment without cardiac arrest), which may represent a group in whom early prone positioning is considered as an adjunct to lung-protective ventilation when oxygenation is impaired. To facilitate future comparisons, detailed reporting of key variables - such as initial blood gas values, time from rescue to intubation, resuscitation details, and ventilator settings - remains important [2].

This report has several limitations. First, the sample size was small, and the retrospective, descriptive design does not allow causal inference. Second, ventilator settings and other co-interventions were not standardized; therefore, changes in P/F ratio and compliance may have been influenced by concurrent adjustments (e.g., FiO_2_ and PEEP) and by the natural course of drowning-associated lung injury. Third, we did not compare outcomes with a control group managed without prone positioning. Accordingly, our findings should be interpreted as hypothesis-generating.

Nevertheless, the short-term increases in oxygenation and respiratory mechanics observed shortly after initiation highlight the need to evaluate prone positioning in pediatric drowning without cardiac arrest in larger, standardized studies. Prospective observational studies using standardized lung-protective ventilation are needed to examine proning responsiveness (e.g., P/F ratio, Cdyn/kg) and its relationship with clinically meaningful outcomes.

## Conclusions

In three pediatric patients with acute respiratory failure after non-cardiac-arrest drowning, early short-duration prone positioning was followed by short-term improvements in oxygenation (P/F ratio) and weight-normalized dynamic respiratory system compliance (Cdyn/kg) within four hours. Because this was a small retrospective case series, causal relationships cannot be established, and the observed changes may have been influenced by concurrent bedside management. These findings are therefore descriptive and hypothesis-generating, and prospective multicenter studies with standardized ventilator management are needed to clarify indications, optimal timing and duration, and associations with patient-centered outcomes.
